# Co-Digestion of Grape Marc and Cheese Whey at High Total Solids Holds Potential for Sustained Bioenergy Generation

**DOI:** 10.3390/molecules25235754

**Published:** 2020-12-06

**Authors:** Josue Kassongo, Esmaeil Shahsavari, Andrew S. Ball

**Affiliations:** School of Science, RMIT University, Melbourne, VIC 3083, Australia; esmaeil.shahsavari@rmit.edu.au (E.S.); andy.ball@rmit.edu.au (A.S.B.)

**Keywords:** anaerobic digestion, cheese whey, electrical conductivity, grape marc, methane production, salinity

## Abstract

At the end of fermentation, wine contains approximately 20% (*w*/*v*) of solid material, known as grape marc (GM), produced at a yield of 2 t/ha. Cheese manufacture produces cheese whey (CW), which is over 80% of the processed milk, per unit volume. Both waste types represent an important fraction of the organic waste being disposed of by the wine and dairy industries. The objective of this study was to investigate the bioenergy potential through anaerobic codigestion of these waste streams. The best bioenergy profile was obtained from the digestion setups of mixing ratio 3/1 GM/CW (wet weight/wet weight). At this ratio, the inhibitory salinity of CW was sufficiently diluted, resulting in 23.73% conversion of the organic material to methane. On average, 64 days of steady bioenergy productivity was achieved, reaching a maximum of 85 ± 0.4% CH_4_ purity with a maximum cumulative methane yield of 24.4 ± 0.11 L CH_4_ kg^−1^ VS. During the fermentation there was 18.63% CODt removal, 21.18% reduction of conductivity whilst salinity rose by 36.19%. It can be concluded that wine and dairy industries could utilise these waste streams for enhanced treatment and energy recovery, thereby developing a circular economy.

## 1. Introduction

Traditionally, lignocellulosic-rich biomass is resistant to natural degradation and therefore difficult to utilise. For example, grape marc (GM), a residue consisting of grape seeds, skins, and stalks, often represents a disposal problem for wineries [1,2,3]. The Australian wine industry has a 4% market share of the global wine industry, and the 5th largest wine exporter, crushing 1.73 million tonnes of wine grapes in 2019 alone [4]. Depending on the grape variety, moisture content, and juice extraction method used, the subsequent byproduct wastes can reach as much as 27% average of the initial fresh weight [5]. This poses a secondary treatment concern and waste removal challenge among large wine producers. Consequently, this business segment has been outsourced to industry players specialising in value-creation from such wastes. In Australia, Tarac Technologies oversees operations for the collection of grape marc from at least 90% of the wine industry [6].

To address this environmental issue, various treatment strategies utilising winery wastes as feedstock have been trialled. These include extraction of commercially relevant chemicals such as ethanol, phenol, and tannin, the composting of GM and its application in animal feeds, and the use of GM as a substrate in energy-conversion technologies including pyrolysis, gasification, and combustion [7,8,9,10]. In the biological treatment of GM, Javier et al. [11] achieved mono-digestion in “wet” reactor setups through progressive activation and acclimatisation. Previously, Makadia et al. [12] proceeded through the codigestion of grape marc with other organics in “wet” anaerobic digestion (AD) systems for methane generation. There are indications that the optimisation of energy recovery utilising GM as a substrate in anaerobic systems holds the potential for valorisation of this abundant sustainable resource [13]. However, energy recovery through any of the thermal conversion processes mentioned above (viz. gasification) are highly endergonic reactions and polluting [10]. In contrast, microbial-mediated processes for bioenergy extraction exhibit greater efficiencies, although cumulative energy outputs vary among studies due to differences in biocatalysts, organic substrate, pretreatment, and digestion conditions used [14]. Therefore, continued research aimed at technology improvements is warranted.

The industrial production of cheese from milk processing results in over 80% of watery waste cheese whey [15,16]. In 2018, Australia had 6% of the worldwide dairy marketplace, with cheese exports of 2.4 million tonnes [17]. Considering the average cheese/whey ratio of 20/80 per cubic volume of fresh milk in the cheese-making operations, the dairy industry is thus confronted with a major problem area for waste management. This liquid fraction is highly polluting due to its high chemical oxygen demand [18]; in addition, high ammonia concentration may result in biological treatment failure [19]. Initial efforts for the valorisation of CW often include the isolation of important building blocks such as lactose, proteins, and minerals [20]. Downstream energy-geared technologies often digest CW in conjunction with other substrates for optimally combined waste biodegradation [21,22,23]. There is a vast array of organic substrates that can be codigested with CW in the determination of the most promising prospective bioenergy application. For conventional thermal conversion methods production feedstock with less than 10% moisture are required [24]. Therefore, CW would require significant pretreatment before use in such thermal applications, considering the water content is generally above 90% [25]. Consequently, anaerobic digestion utilising biocatalysts has consistently been implemented to utilize these problematic wastes [9,14].

Anaerobic treatment processes are broadly divided into two types depending on the solids load contained in the reactor. Solid-state anaerobic digestion (SS-AD) with a total solids loading ≥10% of working volume contrasts with bubbled liquid AD operated at ≤10% total solids [26]. SS-AD essentially offers greater feedstock utilisation, lower water addition, and higher biodegradation levels. Despite these advances, SS-AD reactors have faced operational issues related to a lack of digestate homogeneity, rapid acidification, and inadequate heat and mass transfers [8,27,28]. In contrast, liquid (“wet”) reactor systems have enjoyed extensive detailed studies, mathematical model development and routine full-scale implementation. To illustrate, a waste-to-energy plant built by Yarra Valley Water north of Melbourne, Australia, was commissioned for a treatment capacity of 33,000 tonnes of organic wastes in 2017 [29]. This energy-generation facility fully meets the energy requirements for the nearby sewage treatment plant; surplus electricity is exported to the grid.

However, liquid digestions require substantial reactor sizes, large volumes of water and additional financial costs for continual sludge heating and mixing [29,30]. In contrast, SS-AD systems have proven more effective in the treatment of lignocellulosic material than their liquid-based counterparts [31,32,33]. One explanation is that the characteristic solid organic matrices of SS-AD systems, including humidity content, closely mimic the natural habitat of microorganisms for growth and metabolism [34]. In addition to providing adhesion surfaces, lignocellulosic matrices achieve higher contact of microorganisms with substrates during dry fermentation.

When investigating the impact of temperature on reactor performance, Shi et al. [35] concluded that thermophilic temperatures are better suited for the treatment of lignocellulosic biomass than the mesophilic temperature range; faster reaction kinetics and greater waste reduction occurred at higher temperatures. Forster-Carneiro et al. [36] also established that thermophilic temperatures were appropriate for the biodegradability of solid organic wastes.

To ameliorate the negative environmental impact of untreated wastes from wine- and cheese-making processes, one sustainable remediation strategy would be the establishment of a biodegradable codigestion using lignocellulosic GM with CW wastes. Previously, both Kassongo et al. and Togo et al. [37,38] concluded that CW was a promising substrate in submerged fermentation for electricity generation through microbial fuel cell technology. However, to the best of the authors’ knowledge, the direct codigestion of GM and CW in SS-AD systems for bioenergy production has not been previously reported. 

In this context, this study aimed to investigate the potential for methane production at thermophilic temperature by codigesting GM and CW in defined ratios, resulting in differential dilutions while simultaneously varying the total solids (TS) content. Anaerobic monodigestion of GM was also evaluated to assess the impacts of codigestion in terms of enhanced methane production.

## 2. Results and Discussion

### 2.1. Biogas Production

#### 2.1.1. Cumulative Biogas Yield with Various Mixing Ratios

**Figure 1:**
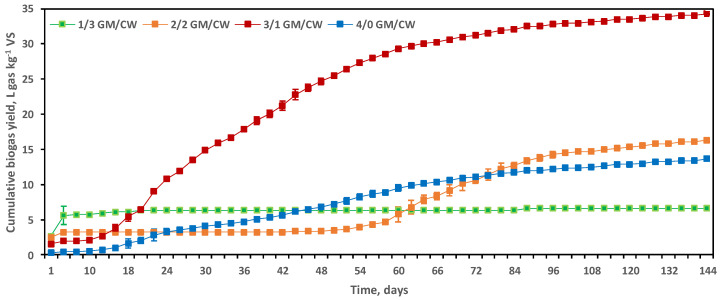
Cumulative biogas yield (L gas kg^−1^ VS) in dry-thermophilic digesters (55 °C). Triplicate digesters were averaged and reported as mean ± standard error. The separate feedstock mixing ratios were 1/3 GM/CW (*w*/*w*; green); 2/2 GM/CW (*w*/*w*; orange); 3/1 GM/CW (*w*/*w*; red); and 4/0 GM/CW (*w*/*w*; blue).

After a 10-day lag period, digesters containing 3/1 GM/CW (*w*/*w*) produced cumulative biogas of 34.24 ± 0.1 L gas kg^−1^ VS, exhibiting a predominantly monophasic curve (Figure 1).

Replicate digesters containing 1/3 GM/CW (*w*/*w*) were characterised by a cumulative 6.60 L gas kg^−1^ VS. Biogas production peaked on day 2 before slowing down, with production remaining low for most of the fermentation. Biogas production reached a cumulative 16.31 ± 0.2 L gas kg^−1^ VS in digesters containing 2/2 GM/CW (*w*/*w*) following a lag of 54 days (Figure 1). This low biogas production was likely due to inhibitory effects of protein-rich CW, which is known to result in the build-up of ammonia when used as a predominant component of a codigestion [39].

Cumulative biogas yield in the GM monodigestion digesters containing 4/0 GM/CW (*w*/*w*) were initially the lowest; performance later improved, reaching a cumulative 13.66 ± 0.1 L gas kg^−1^ VS (Figure 1).

#### 2.1.2. Cumulative Specific Methane Yield (SMY)

**Figure 2:**
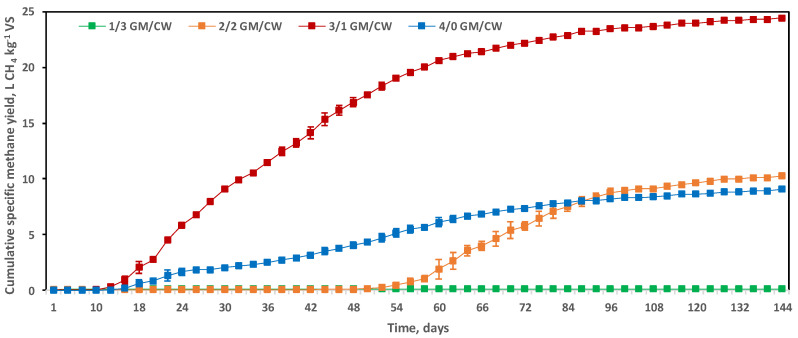
Cumulative methane production (L CH_4_ kg^−1^ VS) in digesters containing 1/3 GM/CW (*w*/*w*) [green]; 2/2 GM/CW (*w*/*w*; orange); 3/1 GM/CW (*w*/*w*; red); and 4/0 GM/CW (*w*/*w*; blue) at 55 °C. Values reported as mean ± standard error.

The experimental SMY in digesters containing 3/1 GM/CW (*w*/*w*) reached 24.43 ± 0.11 L CH_4_ kg^−1^ VS, the highest yield obtained (Figure 2). The calculated theoretical SMY was 103 L CH_4_ kg^−1^ VS, i.e., 23.73% of the organic C was converted to CH_4_. In contrast, the cumulative SMY reached only 9.08 ± 0.1 L CH_4_ kg^−1^ VS in digesters containing 4/0 GM/CW (*w*/*w*), corresponding to 12.87% bioconversion of organic C to CH_4_. When compared to other GM-based studies, higher digestibility has been reported, depending on the wine production methods employed and the grapevine cultivars used. Javier et al. [11] using GM wastes as a sole substrate achieved an average biodegradability of 51%, whilst Fabbri et al. [40] reported that the GM biodegradation index could reach 71%.

The lag phase is an important parameter of the efficiency of AD [13]. In digesters containing 3/1 GM/CW (*w*/*w*) both cumulative gas yield and methane production rate were the greatest. In these digestions, the lag to methane production was 10 days preceded by an overproduction of CO_2_, which gradually decreased. The digesters produced increasingly greater daily volumes of methane during steady-state biogas production over 64 days before declining (Figure 2).

Methanogenesis inhibition occurs when there is accumulation of volatile fatty acids (VFAs) during acidogenesis, coupled to subsequently slower downstream consumption of the metabolites by microorganisms [41]. While evaluating the effect of substrate concentration on dry mesophilic anaerobic digestion of the organic fraction of municipal solid waste (OFMSW), Fernandez et al. [42] found that an increase in solids content to 30% TS for digestion required 0–35 days for hydrolysis and acidogenesis to occur, resulting in a high concentration of fatty acids (up to 1.254 g L^−1^ for acetate). Acetate is the primary precursor for acetoclastic methanogenesis in AD. The balance of metabolite production and removal rates will thus result in either activation or inhibition of downstream catalytic reactions in the digestate. In another study that dealt with OFMSW, methane production was observed only from day 63 [43].

The cumulative SMY in digesters containing1/3 GM/CW (*w*/*w*) was 0.13 L CH_4_ kg^−1^ VS (Figure 2). Based on the experimental CODt removed, the theoretical methane production was 23.33 L CH_4_ kg^−1^ VS; only 0.56% of organic carbon was converted to methane gas. The initially poor digestibility of the organic fraction in digesters containing 1/3 GM/CW (*w*/*w*) may be a contributory factor to the relatively low SMY obtained.

The addition of comparable amounts of GM to CW to lower the dilution of digesters containing 2/2 GM/CW (*w*/*w*), potentially mitigating the inhibitory effects of protein-rich CW resulted in improved process stability, with SMY that accrued to 10.27 ± 0.2 L CH_4_ kg^−1^ VS with an associated 30-day steady-state. The theoretical SMY, based on CODt removal was 159 L CH_4_ kg^−1^ VS, with the conversion of 12.58% of organic C to methane. Similarly, Dinuccio et al. [44] concluded that considerable lignocellulosic material was undegraded during AD because of its crystalline structure.

Makadia et al. [12] only achieved a total SMY of 31 L CH_4_ kg^−1^ VS in the codigestion of milled GM and winery wastewater at 35 °C over 15 weeks despite the application of a liquid-based digester system with regular mixing. In addition, Rebecchi et al. [45] monodigested GM in 0.05 L “wet” systems at 55 °C over 12 weeks, resulting in a cumulative SMY of 40 L CH_4_ kg^−1^ VS. Taken together, irrespective of GM composition, digestion temperatures and digester configurations, the application of GM in batch systems had similar bioenergy outputs. However, SS-AD in our study, especially the ratio of 3/1 GM/CW (*w*/*w*) had greater organic load per cubic volume of the reactor because of the high TS, allowing for more feedstock treatment. In addition, the lack of mixing was an additional cost-saving step, whilst still achieving competitive calorific productivity within the same feedstock type.

Da Ros et al. [13] demonstrated that GM as feedstock for larger treatment capacity in 5-L continuously stirred systems were capable of exceeding 300 L CH_4_ kg^−1^ VS and that temperature increase did not enhance digestion. However, there would be additional costs in continuously mixing and heating such “wet” systems [29]. Therefore, the increased experimental SMY values reported by Da Ros et al. [13] is not necessarily a guarantee of a system’s economic viability as the bioenergy recovered must be considered alongside the combined specific energy requirements for methane production [41]. To bridge this gap, energy balance analyses allow for adequate appraisal across studies.

### 2.2. Physicochemical Characteristics of Effluent

#### 2.2.1. pH

The inhibition of methanogenesis observed in codigestion experiments was accentuated by a pH reduction, likely due to the release of phenolic compounds and organic acids causing a gradual acidification of the digestate [11,46,47,48]. The initial pH, 7.20 decreased to 5.79 and 6.44 in digesters containing 1/3 GM/CW (*w*/*w*) and 2/2 GM/CW (*w*/*w*), respectively (Table 1).

In addition, methanogens are known to be sensitive to H^+^ levels in the digestate and thus possess a narrow optimal pH range, 6.6–7.2 [49]. Digesters containing 3/1 GM/CW (*w*/*w*) had an alkaline starting pH (8.52) that slowly increased over time with methane production over a prolonged operation, in contrast to digesters that were already near pH 7 at the start.

Moreover, the optimal pH for ammonification is between pH 6.5 and 8.5 [50]. Digesters with an initial pH of 7.2, containing diluted CW setups did not hold sufficient buffering strength for prolonged digestion. This may explain the pH reduction to as low as pH 5.79 in these digesters. However, at lower CW-based dilution (e.g., 3/1 GM/CW and 4/0 GM/CW), effluent pH was within the optimal range (Table 1).

The pH was stable at 8.21 ± 0.1 in GM monodigestion digesters, aided by a lack of exogenous CW-related proteins. Moreover, nitrate ammonification may have outcompeted denitrification because of the initially high carbon (i.e., electron donor) and low NO_3_^−^ (i.e., electron acceptor) concentrations together with thermophilic conditions [51,52].

#### 2.2.2. Electrical Conductivity

Hydrolysis of polymers such as carbohydrates, proteins, and lipids into their respective monomers lowers electrical conductivity in the extracellular medium, thus diluting conductive ionic species, which shuttle electrons, which are required for microbial metabolism and growth (Table 1; [53]). Klein et al. [54] monitored the intrinsic conductivity of grapevine residues in anaerobic digesters over 26 weeks; an initial conductivity of 15 mS cm^−1^ yielded 0.11 m^3^ CH_4_ kg^−1^ VS; this declined to 0.04 m^3^ CH_4_ kg^−1^ VS when conductivity reduced to 10.1 mS cm^−1^. Changes in conductivity were shown to approximate methane yield up to 2 days ahead of biogas production. Therefore, the magnitude of variation in the electro potential parameter can be related to the rate of hydrolysis. This was confirmed in the results from the digesters containing 3/1 GM/CW (*w*/*w*), which showed the least reduction in conductivity (21.18%) and the highest SMY; this contrasted with digesters containing 1/3 GM/CW (*w*/*w*), which had the highest change in conductivity (89.84%) and the lowest SMY.

Through an understanding of the benefits of conductivity for AD, various studies have improved bioenergy profiles through supplementation with exogenous conductive materials such as granular activated carbon, stainless steel, magnetite, and iron powder, among others [55,56,57,58]. However, synthetic conductive materials are expensive to produce and are confirmed environmental hazards, thus limiting their widespread applications [59]. Therefore, enhancement of microbe-driven conductivity control would be better suited for sustainable biogas productivity.

#### 2.2.3. Salinity

Salinity is an important factor in anaerobic digestion. As shown in Table 1, digesters containing mostly CW had high salinity, which could be detrimental to adequate biogas production. However, the codigestion ratio 3/1 GM/CW (*w*/*w*) provided a favourable initial salinity of 5.25% ± 0.4%, which increased to 7.15% ± 1.4% by the end of the treatment (Table 1). Low salinity may trigger increased hydrolysis and acidification, which may ultimately lower methane production [60]. This was evidenced by the monodigestion of 4/0 GM/CW (*w*/*w*), which exhibited an inhibited SMY profile, despite an initial low salinity of 7.00% ± 0.1%. In contrast, methane production was mostly hindered in digesters at higher dilutions (1/3 GM/CW and 2/2 GM/CW) where salinity was initially elevated. Previous studies concluded that feedstock codigestion was a cost-effective strategy in the control of salinity to improve SMY [61,62].

Polymers such as carbohydrates and proteins are generally bound in granular states before AD. An optimal Na^+^ concentration of 0.23–0.35 g L^−1^ for microbial metabolism leads to solubilisation of polymers, stimulating the production of short-chain fatty acids, which in turn promote acetate production for subsequent methanogenesis. However, excessive salinity reduces the biodegradability of acetate and alters the osmotic pressure of the digestate, causing loss of cellular integrity and reduction in enzymatic activities among methanogens [60]. In addition, methane production can be negatively impacted by the high concentration of sodium chloride, which inhibits the rate-limiting hydrolysis step and acidification stage [62,63]. In exploring the effect of 0–15.0 g L^−1^ salinity on anaerobic mono- and codigestion of food wastes, Zhao et al. [60] established a strong correlation between increasing Na^+^ concentration and inhibition of methane production resulting in extended digestion times. Similarly, Rinzema et al. [64] noted gradual to complete inhibition of SMY with Na^+^ 5–14 g L^−1^ in granular UASB reactors.

Taken together, interspecies electron transfer conducive to an optimal biogas profile requires the least possible disturbance in the electrodynamics of the digestate. Noteworthy, only setups 3/1 GM/CW (*w*/*w*) had a salinity rise of 36.19% (Table 1). The initial salt concentration (5.25%) was adequate to promote solubilisation of polymers with resulting metabolite intermediates utilised by downstream methanogens. The increased hydrolysis continually released ionic species and soluble minerals, which were initially in the granular state before digestion, biochemically controlling the loss of specific conductance and further raising salinity in a positive feedback loop [60].

### 2.3. Nutrition

Biochemical pathways naturally convert organically bound nitrogen into ammonia, which is readily assimilated by the growing microbial population. Total Kjeldahl nitrogen (TKN) represents the sum of ammonia-nitrogen with organic-bound nitrogen [13,65]. The COD/N ratio in the effluent was thus used to approximate the nutritional quality of the digestate during reactor cycles. The COD/N ratios ranged between 17.05/1 and 50.58/1 in the digesters (Table 1). A low COD/N usually results in free ammonia inhibition due to the overabundance of nitrogen. Taken together, the highest COD/N ratio was observed in digesters containing 3/1 GM/CW (*w*/*w*); this resulted in an increased methane yield [66].

A 12.82% increase in soluble COD (CODs) was observed in digesters containing 3/1 GM/CW (*w*/*w*), attributable to an increase in particulate COD being digested and thus raising the soluble fraction. However, plant cell walls, especially those from the stalks and seeds of GM are composed of lignin and cellulose, which are resistant to degradation [44]. However, CODs, composed of readily biodegradable sugars and alcohols were removed from the digestate, resulting in the reduction of CODs in most digesters. Irrespective of codigestion set up, the total COD (CODt) removed during the treatment of the winery residues was low in comparison to treatment efficiencies generally achieved in the literature for other waste types such as organic fraction municipal solid waste (OFMSW), which can reach as high as 83% CODt removal under thermophilic conditions [67]; this difference, confirms to the recalcitrance of GM [40]. After anaerobic treatment, solid wastes can be used as an agricultural soil amendment due to the improved agronomic potential of the digestate [29]. As evidenced in Table 1, digesters containing 3/1 GM/CW (*w*/*w*) had an enriched physicochemical composition for microbial growth. Nevertheless, additional phytotoxic studies may be required before land applications [68].

Suitably mixed feedstocks are known to exhibit higher bacterial enzyme activities and higher gas production efficiencies than monodigestion setups alone due to improved nutritional balance along with additional microbial symbioses [60]. This behaviour was supported by an increased rate of methanogenesis whilst biogas production remained relatively stable (Figure 2, slope of digesters containing 3/1 GM/CW (*w*/*w*)). Furthermore, monodigestion digesters containing 4/0 GM/CW (*w*/*w*) offered a baseline reactor performance in order to determine the effect of CW addition. Following wine production and further distillation of solid residues for secondary alcohol extraction, discarded winery wastes can be diverted to anaerobic treatment. An intercalated step for watery CW addition to achieve bioaugmentation, moisture control, and nutritional improvement would fulfil the formation of a circular economy jointly backed by the wine and dairy industries in the framework of the Millennium Development Goals [29].

The substantial reduction in TS and VS concentrations within treatment setups did not result in methane production (Table 1). This behaviour may be governed by the recalcitrant nature of lignocellulosic material of large organic particle sizes conducive to slow hydrolysis, followed by slow metabolite utilisation [69]. Kim et al. [47] established that during AD, CODt removal is repressed due to the hydrolysates not readily converted to VFAs, indicating that the acidogenesis was rate-limiting. Our study exhibited CODt trends that were generally stable between the start and the termination (*p* > 0.05) of the respective digestions, thus confirming inhibition of the fermentation stage.

### 2.4. Regression Models for Data Fit

**Figure 3:**
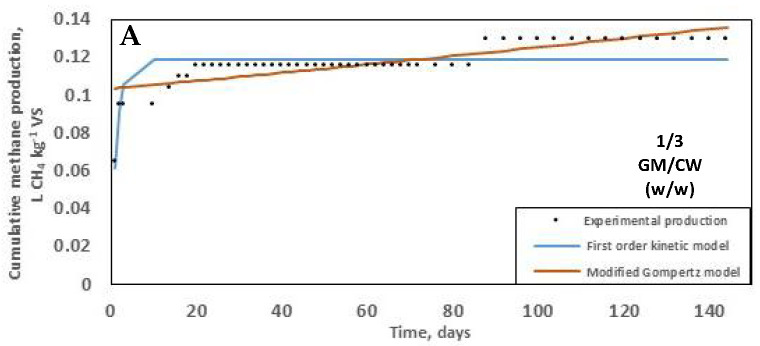

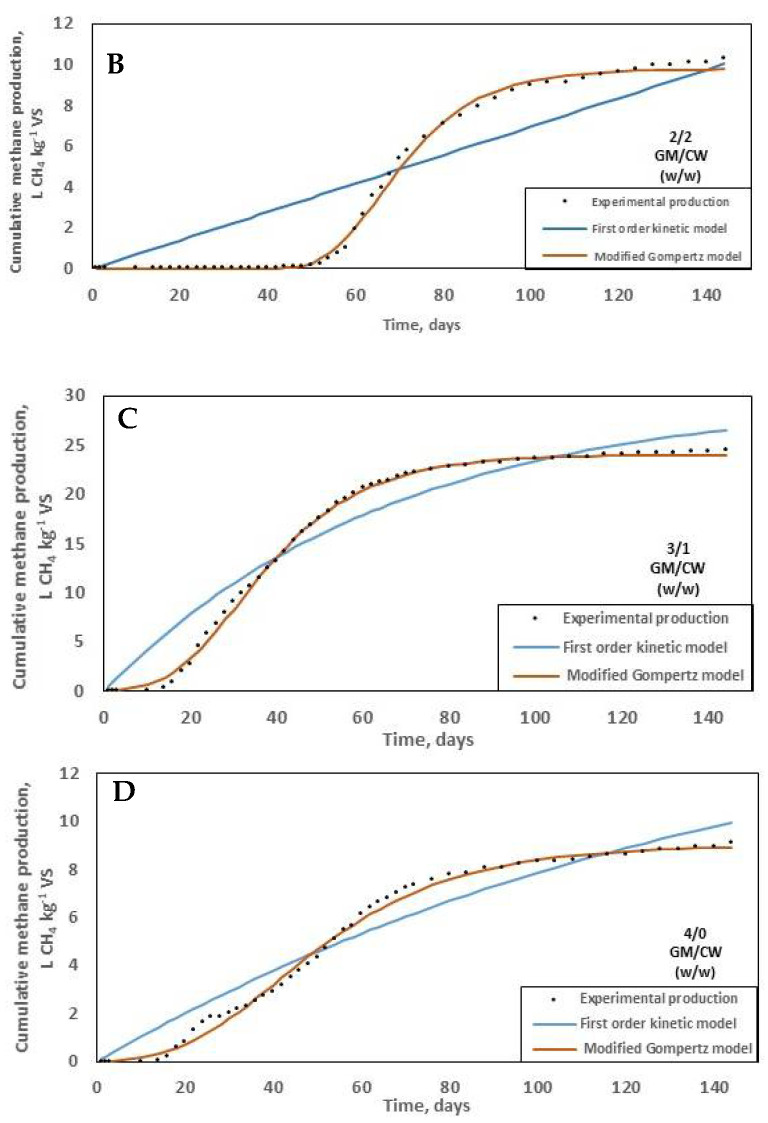
Plot of simulated predictive cumulative methane yield (L CH_4_ kg^−1^ VS) against experimental values obtained using first-order linear model (blue) and the modified Gompertz model (orange) for digesters containing: 1/3 GM/CW (*w*/*w*) (**A**); 2/2 GM/CW (*w*/*w*) (**B**); 3/1 GM/CW (*w*/*w*) (**C**); and 4/0 GM/CW (*w*/*w*) (**D**).

A kinetic study was carried out by fitting both the first-order and the modified Gompertz models to the experimental data. The predictive parameters and corresponding values of the simulations are shown in Table 2. Comparative analyses of models fitted using experimental SMY values showed that the modified Gompertz better described the data in all instances (Figure 3). In addition, the critical test statistic sum of squared deviations (SSDs) showed lower fluctuation in the modified Gompertz model than in the first-order kinetic model within digesters containing the same mixing ratio, indicating the robustness of the model fit for the particular digestion setups (Table 2). For example, SSD values were 287.68 and 6.69 in the first-order and the modified Gompertz models, respectively, in digesters containing 3/1 GM/CW (*w*/*w*) confirming that the modified Gompertz model was more applicable for the description of reaction kinetics in digesters containing 3/1 GM/CW (*w*/*w*; Figure 3C).

The modified Gompertz model is generally applicable in scenarios where methane production requires an acclimation period characterised by a lag phase. Compounds such as alcohol and phenol, present in winery residues require longer incubation periods before digestibility, thus explaining the regularity of lag phase in the setups (Figure 3; [70]). Additionally, poor waste digestibility with an associated protracted lag time often results from the anaerobic treatment of grape seeds commonly found in winery wastes [71].

## 3. Materials and Methods

### 3.1. Pretreatment and Analytical Methods

Spent GM that had undergone prior distillation for alcohol recovery was sourced from Tarac Technologies, Nuriootpa, Australia. The sludge inoculum was sampled from Melbourne Water, Melbourne, Australia. Reactants were stored at 4 °C until use. The characterisation parameters reported were conducted in triplicate on samples before (Table 3) and after digestion (Table 4). Solids, COD and the total Kjeldahl nitrogen (TKN) were determined according to standard methods [72]. Conductivity and salinity were determined with the use of a compact conductivity meter (LAQUAtwin-CC-11, HORIBA Scientific) and a compact salt meter (LAQUAtwin-Salt-11, HORIBA Scientific, Kyoto, Japan), respectively.

Grape marc and cheese whey were mixed in the following ratios: 1/3 GM/CW (*w*/*w*); 2/2 GM/CW (*w*/*w*); 3/1 GM/CW (*w*/*w*); and 4/0 GM/CW (*w*/*w*) before digestion over 144 days (Table 4).

### 3.2. Reactor Configuration

Two batch replicate setups of GM and CW of variable mixing ratios were operated in parallel. The substrate-to-inoculum ratio (SIR) was 10:1 for a working volume of 110 mL incubated at 55 °C; the mixing ratios of feedstock were 1/3 GM/CW (*w*/*w*), 2/2 GM/CW (*w*/*w*), 3/1 GM/CW (*w*/*w*), and 4/0 GM/CW (*w*/*w*). The headspace volume within the standard 250 mL Pyrex^®^ glass reaction bottles (SciLabware Limited, Staffordshire, UK) was 200 mL. The pH was adjusted to 7.2, with use of H_2_SO_4_ and NaOH stock solutions, in mixing ratios 1/3 and 2/2 GM/CW (*w*/*w*) to strengthen long-term buffering. A HANNA Instruments edge^pH^ was used to measure pH. Daily biogas volumetric production was measured using water displacement [13,73]. The biogas composition was measured in a GEM2000 Landfill Gas Analyser (Geotech, Coventry, UK).

### 3.3. Biogas Study

#### 3.3.1. Specific Methane Yield

The specific methane yield (SMY) of each digestion setup corresponded to the cumulative methane fraction of the cumulative biogas expressed as a function of the VS_fed_, as digestion progressed. Replicate setups of corresponding mixing ratios were averaged and reported as mean ± standard error values. SMY is expressed as L CH_4_ kg^−1^ VS [74,75].

#### 3.3.2. COD-Equivalents

The biodegradability or biodegradation degree for samples corresponds to the amount of COD removed through methane production. The COD equivalence was calculated on the basis that 350 mL of methane production corresponds to 1 g of COD removed from digestion. COD-equivalents can thus be expressed in kg COD kg^−1^ VS [74,75]. The theoretical assumption is that COD is completely digested for methane production without accounting for microbial metabolism [13].

### 3.4. Statistical Treatment

One-way analyses of variance (ANOVA) of methane production for digestion setups at the 0.05 significance level was conducted. Mean values were separated using Tukey’s HSD test, where the F-value was significant for the difference between the means of physicochemical indicators for treatment setups (Mini version s33d25, http://www.statskingdom.com, Statistics Kingdom).

### 3.5. Kinetic Simulations

To describe the methanation process, non-linear regressions were utilised [40,76]. The degradation of organics were assumed to be patterned along a first-order rate of decay due to the microbial role in the fermentation process, thus the first-order Equation (1):

*B*(*t*) = *B*_0_ (1 − exp(−*kt*)) (1)

where *B*(*t*) is the cumulative methane volume (L CH_4_ kg^−1^ VS) at a digestion time *t*(d); *B*_0_ is the methane potential of the substrate material (L CH_4_ kg^−1^ VS); *k* is the first-order disintegration rate constant (d^−1^); and *t* is the digestion time (d).

In order to estimate the lag phase, the modified Gompertz model was simulated Equation (2):

*B*(*t*) = *B_0_* exp{−exp[(*R_m_* exp/*B_0_*) (*λ* − *t*) + 1]} (2)

where *R_m_* is the maximal methane production rate (L CH_4_ kg^−1^ VS d^−1^) and *λ* is the lag phase (d); all mathematical models were simulated with the Solver tool of Microsoft Office Excel.

## 4. Conclusions

In this study, we demonstrated the bioenergy potential of grape marc, a dense lignocellulosic feedstock routinely retrieved from winery-related activities. Both the monodigestion of grape marc and the codigestion of grape marc along with cheese whey resulted in detectable biogas production, albeit at different levels. The sustained methane production at high total solids in unmixed conditions benefited from sufficient buffering of grape marc without requirements for pH adjustment. However, methanogenesis inhibition was observed in setups with predominant protein-rich cheese whey.

The results of this study enhanced the understanding of the feasibility of successful anaerobic treatment and bioenergy generation using recalcitrant agro-industrial wastes at elevated solids concentration without expensive waste amendment. We established that the codigestion of grape marc and cheese whey in 3/1 ratio (*w*/*w*), respectively, was optimal for solid-state anaerobic digestion. Methane production reached 24.43 ± 0.11 L CH_4_ kg^−1^ VS in unmixed conditions during dry-fermentation. There was a syntrophic relationship between electron donors and downstream electron-accepting methanogens in the digestate, which behaved as an effective conductive material resulting in optimised methane production. A rise in salinity was identified as a necessary outcome to optimise the biodegradability of organics and methane production.

This study digested unaltered waste materials without requirements for clean water for dilution or hazardous artificial conductive materials, routinely employed in parallel studies. The experimental approach aligns with the concept of self-sustainable digestion while improving the energy balance required for methane production. The codigestion of grape marc and cheese whey improved overall feedstock treatment, increased methane output, and compounded the valorisation of individual wastes. This experiment represents the first report documenting the bioenergy potential of fermentation of grape marc and cheese whey in solid-sate anaerobic digestion systems.

Future efforts will explore the impact of treatment capacity on methane yield and the effect of lowering the energy requirements for digestion. These strategies will provide important information regarding how these operational factors affect biogas production during the codigestion of grape marc and cheese whey.

## Figures and Tables

**Table 1:** Effluent characteristics at the completion of the anaerobic treatment. Replicates were configured in a parallel arrangement of grape marc (GM) and cheese whey (CW), on wet weight basis, of ratios 1/3 GM/CW; 2/2 GM/CW; 3/1 GM/CW; and 4/0 GM/CW. Values recorded as mean ± standard error.

Parameter	Unit	1/3 GM/CW	2/2 GM/CW	3/1 GM/CW	4/0 GM/CW
TS	%	1.60 ± 0.3	3.17 ± 3.3	10.5 ± 3.4	19.4 ± 1.7
VS	%	0.91 ± 0.2	1.38 ± 0.3	7.58 ± 2.6	12.1 ± 0.5
CODt	g L^−1^	90.0 ± 10	191 ± 22	214 ± 5.0	219 ± 25
CODs	g L^−1^	27.0 ± 3.0	29.5 ± 3.5	22.0 ± 4.0	20.0 ± 3.0
TKN	g L^−1^	4.60 ± 0.1	8.29 ± 0.2	4.23 ± 0.5	12.8 ± 0.1
COD/N	—	19.57	23.03	50.58	17.05
pH	—	5.79 ± 0.0	6.44 ± 1.0	7.64 ± 0.1	8.21 ± 0.1
EC	mS cm^−1^	9.38 ± 0.0	12.9 ± 0.6	13.4 ± 2.5	10.6 ± 0.3
Salinity	%	6.15 ± 2.3	8.70 ± 2.7	7.15 ± 1.4	3.65 ± 0.1

**Table 2:** Kinetic parameters calculated by the predictive non-linear regression models: first-order kinetic and the modified Gompertz for digesters containing: 1/3 GM/CW (*w*/*w*); 2/2 GM/CW (*w*/*w*); 3/1 GM/CW (*w*/*w*); and 4/0 GM/CW (*w*/*w*).

Simulation	Unit	1/3 GM/CW	2/2 GM/CW	3/1 GM/CW	4/0 GM/CW
**First-order kinetic model**					
*B*_0_	L CH_4_ kg^−1^ VS	0.118993172	3663.316604	29.88610485	16.04597425
*k*	d^−1^	0.721169765	1.90423 × 10^−5^	0.015248799	0.006743193
Sum of squared deviations (SSD)	—	0.003090119	188.8049528	287.6845951	33.82613142
Measured methane yield—day 144	L CH_4_ kg^−1^ VS	0.129553030	10.27407727	24.42713824	9.079883333
Predicted methane yield—day 144	L CH_4_ kg^−1^ VS	0.118993172	10.03137155	26.56078267	9.969417616
Difference between measured and predictive methane yield (in absolute value)	%	8.150993042	2.362311641	8.734729456	9.796758942
**Modified Gompertz model**					
*B*_0_	L CH_4_ kg^−1^ VS	1.565811821	9.784578865	24.03266289	9.011026908
*λ*	d	0.000000000	53.17945245	14.84340725	18.32337505
*R_m_*	L CH_4_ kg^−1^ VS d^−1^	0.000423702	0.292288763	0.550764399	0.148677144
Sum of squared deviations (SSD)	—	0.002618254	2.713528645	6.691658696	2.748511505
Measured methane yield—day 144	L CH_4_ kg^−1^ VS	0.129553030	10.27407727	24.42713824	9.079883333
Predicted methane yield—day 144	L CH_4_ kg^−1^ VS	0.135785248	9.767920054	24.01174037	8.924128388
Difference between measured and predicted methane yield (in absolute value)	%	4.810553244	4.926546741	1.700558883	1.715384878

**Table 3:** Physicochemical composition of unmixed substrates and inoculum before digestion. Values recorded as mean ± standard error.

Parameter	Unit	Grape Marc	Cheese Whey	Inoculum
TS	%	38.7 ± 1.51	7.87 ± 1.02	2.80 ± 0.28
VS	%	24.1 ± 0.54	3.80 ± 0.88	1.93 ± 0.22
CODt	g L^−1^	223 ± 16.3	67.1 ± 0.42	50.9 ± 1.91
CODs	g L^−1^	47.5 ± 12.0	48.0 ± 5.79	30.5 ± 0.35
TKN	g L^−1^	51.8 ± 0.76	11.5 ± 0.16	13.3 ± 0.72
pH	—	9.19 ± 0.01	5.41 ± 0.01	8.47 ± 0.01
EC	mS cm^−1^	15.0 ± 0.20	14.0 ± 0.34	9.25 ± 0.17
Salinity	%	5.20 ± 0.32	13.9 ± 0.11	2.30 ± 0.20

TS, total solids; VS, volatile solids; CODt, total COD; CODs, soluble COD; TKN, total Kjeldahl nitrogen; EC, electrical conductivity.

**Table 4:** Physicochemical characteristics of digester nutrient at start-up. Codigestion setups of grape marc (GM) and cheese whey (CW), on wet weight basis, were of ratios 1/3 GM/CW; 2/2 GM/CW; 3/1 GM/CW; and 4/0 GM/CW. Values recorded as mean ± standard error.

Parameter	Unit	1/3 GM/CW	2/2 GM/CW	3/1 GM/CW	4/0 GM/CW
TS	%	11.3 ± 1.1	17.3 ± 3.3	28.5 ± 1.1	38.7 ± 1.2
VS	%	6.60 ± 1.1	11.0 ± 2.1	17.1 ± 0.2	12.1 ± 0.5
CODt	g L^−1^	94.0 ± 1.5	241 ± 10	263 ± 20	223 ± 12
CODs	g L^−1^	54.5 ± 2.5	58.5 ± 4.5	19.5 ± 5.5	22.0 ± 2.0
TKN	g L^−1^	15.0 ± 0.0	8.03 ± 0.1	2.56 ± 0.5	12.6 ± 1.5
pH	—	7.20 ± 0.0	7.20 ± 0.0	8.52 ± 0.0	9.03 ± 0.1
EC	mS cm^−1^	92.3 ± 2.3	17.3 ± 0.1	17.0 ± 1.0	31.0 ± 0.4
Salinity	%	15.6 ± 0.0	11.9 ± 0.9	5.25 ± 0.4	7.00 ± 0.1

TS, total solids; VS, volatile solids; CODt, total COD; CODs, soluble COD; TKN, total Kjeldahl nitrogen; EC, electrical conductivity.
